# HIV-1 Tat, apoptosis and the mitochondria: a tubulin link?

**DOI:** 10.1186/1742-4690-2-7

**Published:** 2005-02-07

**Authors:** Mauro Giacca

**Affiliations:** 1 Molecular Medicine Laboratory, International Centre for Genetic Engineering and Biotechnology (ICGEB), Trieste, Italy

## Abstract

The Tat protein of HIV-1 is a powerful activator of viral gene expression. Besides this essential function at the HIV-1 promoter, the protein also exerts a remarkable number of other biological activities, among which the induction of cellular apoptosis. Two papers now published in *Retrovirology *provide possible molecular mechanisms for the pro-apoptotic effect of Tat, which involve the cell's microtubular network and the mitochondrial pathway of apoptosis.

## 

Although more than 20 years have passed since the identification of HIV as the cause of AIDS, several essential questions about its pathogenicity remain as yet unanswered. In particular, a central, still unresolved issue is the mechanism underlying the progressive development of immunodeficiency. It is now well established that HIV infection determines a rapid turnover of infected CD4 cells [1,2]; consistent with this finding, multiple molecular pathways triggered by different HIV proteins are known to lead to cell apoptosis [3,4]. However, the capacity of the immune system to regenerate its cells by far exceeds the number of dying HIV infected cells. Thus, the extension of the apoptotic message to neighboring, bystander cells has long been recognized as a potential mechanism sustaining the immunodeficiency that accompanies HIV disease progression [5].

In this context, the finding that the virus-encoded Tat protein is released by the infected cells and can be taken up by neighboring, uninfected cells via an endocytic mechanism [6,7] has long suggested the possibility that some of the bystander apoptotic effects exerted by HIV might be mediated by this protein. Over ten years ago different investigators did indeed show that extracellular Tat can trigger apoptosis in T-cell lines and primary T-cells [8,9]. The classical apoptotic pathway, involving the cell's mitochondria, is regulated by the Bcl-2 family of proteins. This family contains both anti-apoptotic (Bcl-2, Bcl-XL) and pro-apotpotic (Bax, Bid, Bim) members that exert their function primarily at the mitochondrion by either preventing or inducing mitochondrial dysfunction. Upon receiving a death signal, the pro-apoptotic proteins translocate from the cytoplasm to the outer mitochondrial membrane, where they interact with their pro-apoptotic partners. This occurrence is followed by mitochondrial dysfunction, release of pro-apoptotic proteins out of the mitochondrion (among which, a prominent role can be ascribed to cytochrome c), and subsequent caspase activation [10]. One of the cellular events that trigger the mitochondrial pathway of apoptosis is the disturbance of the dynamic formation of microtubules in the cell. This event can be triggered by a variety of microtubule-targeted, tubulin-polymerizing agents (MTPAs), which include paclitaxel (Taxol) and several other anticancer drugs [11]. Following intracellular uptake, MPTAs bind β-tubulin and promote tubulin polymerization, which interferes with the function of the mitotic spindle resulting in mitotic arrest at the metaphase-anaphase transition and subsequent induction of the mitochondrial pathway of apoptosis.

A link between microtubule polymerization and the pro-apoptotic effect of Tat has first been suggested a few years ago in the observation that Tat directly interacts with the αβ-tubulin dimers and polymerized microtubules in the cytoplasm of the cell [12]. The functional consequence of this interaction, which requires the integrity of four amino acids in the conserved Tat core domain, is the stabilization of microtubules and the consequent prevention of microtubule depolymerization. This disturbance in the microtubular network is a powerful inducer of the mitochondrial pathway of cellular apoptosis, an event that is transduced by the pro-apoptotic Bcl-2 relative Bim. These findings supported previous observations that had already shown that Tat causes changes in mitochondrial membrane permeability [13,14] and that it interferes with the polymerization of microtubules [15].

Two papers now published in *Retrovirology *extend the link between the microtubule network, the mitochondrial pathway of apoptosis, and Tat. De Mareuil and coworkers show that Tat enhances tubulin polymerization into microtubules, an effect similar to that exerted by the MTPAs, and physically associates with the polymerized microtubuli [16]. As opposed to paclitaxel, however, Tat only increases the rate of tubulin polymerization while it does not permanently affect the organization of the microtubule network, nor does it blocks cell cycle progression. Most notably, the ability of different Tat variants to induce tubulin polymerization correlates with their capacity to induce apoptosis. Similar to paclitaxel and other microtubuli damaging agents, the pro-apoptotic effect of Tat parallels the induction of cyctochrome c release from the mitochondria, a critical event triggering apoptosis.

The accompanying manuscript by Epie and coworkers describes the identification of a microtubule-associated protein, LIS1, which specifically binds Tat [17]. In the course of a biochemical project entailing the fractionation of T-cell extracts searching for Tat-associated kinases that phosphorylate the C-terminal domain of RNA polymerase II – a known biochemical activity associated to Tat -, these authors found that LIS1 co-purifies with a complex of proteins including one of the CTD kinases, CDK7, its cyclin partner, cyclin H and the MAT1 co-factor. Of note, out of the four purified proteins, only LIS1 directly bound Tat, as shown by GST-pulldown and co-immunoprecipitation experiments, and by the yeast two hybrid assay. LIS1 is known to regulate microtubule dynamics by interacting with dynein and additional components of the dynein motor [18].

What might be the relevance of these findings in the context of HIV-1 infection? They clearly provide a mechanism for CD4 T-cell apoptosis and for the extension of the apoptotic effect to bystander, uninfected cells in the lymph node. Moreover, the interaction of Tat with the microtubular network might explain the occurrence of neuropathogenesis accompanying the progression of HIV disease, since many human neurodegenerative conditions are elicited by a reorganization of the neuronal cytoskeleton [19]. Thus, the disturbance of the microtubular network induced by Tat adds to other potentially pro-apoptotic mechanisms induced by the protein, such as the upregulation of FasL [9], TRAIL [20], Bax [21] and caspase 8 [22] and the downregulation of Bcl2 [21].

As commonly happens in biology, the findings reported in these manuscripts raise more questions than answers. First, the Tat domains involved in the described interactions are different, a surprising finding given the very small size of Tat. This observation might possibly suggest that Tat is part of a large multi-molecular complex associated with the tubular network, making multiple contacts with different proteins. This issue can be experimentally addressed biochemically, or even within the cell, by taking advantage of the biophysical techniques available to investigate protein-protein interactions in vivo [23]. Secondly, the role of LIS1, if any, in the Tat-triggered mitochondrial pathway of apoptosis or in the functions of CDK7 and its partners, with which it unexpectedly co-purifies is unclear. Third, and most importantly, it remains to be seen whether the concentration at which Tat binds tubulin and exerts its pro-apoptotic effects is compatible with the concentration at which the protein is expressed in the infected cells and diffuses to neighboring cells. As a matter of fact, the measurement of the extracellular concentration of Tat still remains a holy grail in the HIV research field [24], partly due to the weak avidity of the currently available anti-Tat antibodies, partly because of the biological property of extracellular Tat that is sequestered by extracellular matrix proteoglycans [25]. Until more reliable methods are developed to determine the levels of extracellular Tat in vivo, the full biological implications of Tat-induced apoptosis cannot be entirely appreciated.

## Abbreviations

MTPAs: microtubule-targeted, tubulin-polymerizing agents

CTD: carboxy-terminal domain

## Competing interests

The author(s) declare that they have no competing interests.

**Figure 1:**
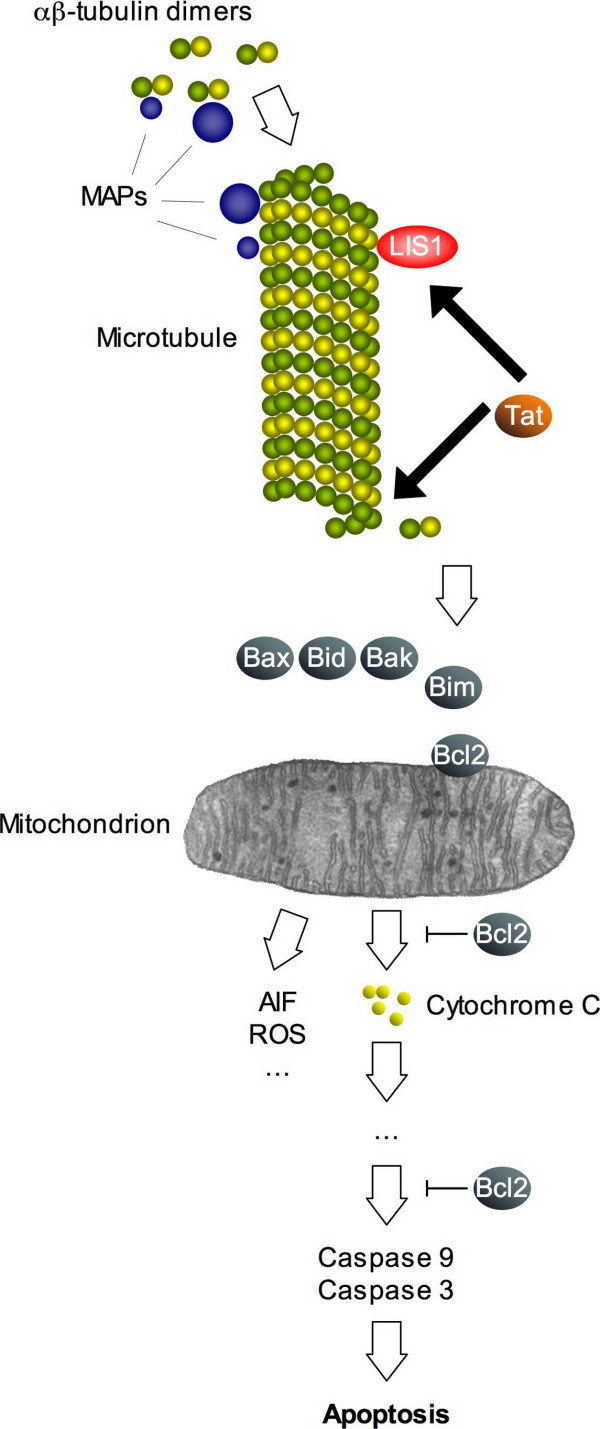
To form microtubules, α- and β-tubulin molecules join to form a heterodimer. These dimers then attach to other dimers forming oligomers that elongate into protofilaments; eventually, the oligomers will join to give rise to a ringed microtubule. Microtubules or unpolymerized tubulin bind microtubule-associated proteins (MAPs), which regulate polymerization, facilitate assembly, stabilize the microtubules and regulate microtubular transport of macromolecules and vesicles. The HIV-1 Tat protein binds to αβ-tubulin dimers and microtubules thus enhancing microtubule polymerization, and to the microtubule-associated protein LIS1, which is also known to facilitate assembly of microtubules. Disturbance of the dynamics of microtubular network formation activates the intrinsic mitochondrial apoptotic pathway. Pro-apoptotic Bcl2 family members – in particular, Bim – are recruited to the mitochondrion; as a consequence, the mitochondrial membrane potential collapses, and pro-apoptotic factors are released into the cytoplasm. These include reactive oxygen intermediates (ROIs), apoptosis-inducing factor (AIF), and cytochrome c, among others. Release of cytochrome c is a point of no return as it leads to autoactivation of caspase 9, which in turn proceeds to cleave the downstream effector caspases (caspase 3, 6, etc.).
